# Active Peyronie’s disease as an early antifibrotic treatment window: a translational review

**DOI:** 10.1093/sexmed/qfag068

**Published:** 2026-08-06

**Authors:** Marek Broul, Michaela Liegertová, Aneta Hujová, Michal Vostrý

**Affiliations:** Department of Sexology, Krajská zdravotní, a.s. - Masaryk Hospital in Ústí nad Labem, o.z., Ústí nad Labem 400 11, Czech Republic; Department of Urology, Krajská zdravotní, a.s. - Litoměřice Hospital, o.z., Litoměřice 412 01, Czech Republic; Faculty of Health Studies, Jan Evangelista Purkyně University in Ústí nad Labem, Ústí nad Labem 400 96, Czech Republic; Faculty of Science, Jan Evangelista Purkyně University in Ústí nad Labem, Ústí nad Labem 400 96, Czech Republic; Faculty of Health Studies, Jan Evangelista Purkyně University in Ústí nad Labem, Ústí nad Labem 400 96, Czech Republic; Faculty of Health Studies, Jan Evangelista Purkyně University in Ústí nad Labem, Ústí nad Labem 400 96, Czech Republic

**Keywords:** Peyronie’s disease, penile induration, fibrosis, myofibroblasts, tadalafil, tamoxifen, phosphodiesterase 5 inhibitors, selective estrogen receptor modulators, patient-reported outcome measures, penile traction

## Abstract

**Introduction:**

Peyronie’s disease (PD) is an acquired fibrotic disorder of the tunica albuginea that can cause pain, curvature, deformity, erectile dysfunction, and substantial psychosexual burden. Management is usually guided by disease phase, yet active disease remains inconsistently defined, and no oral regimen has shown high-certainty disease-modifying efficacy. We synthesize clinical, mechanistic, guideline, outcome-measure, traction, and public-data evidence relevant to active or progressive PD as a target for early antifibrotic trials.

**Methods:**

We conducted a targeted narrative translational review of full-text sources on active-phase definitions, PDE5 inhibitor/selective estrogen receptor modulator (SERM) pharmacology, tadalafil-based clinical studies, placebo-controlled tamoxifen evidence, current recommendations, non-surgical evidence reviews, patient-reported outcome measures, traction therapy, and PD transcriptomic or single-cell studies. We selected sources through targeted database searching, citation chasing, and full-text evidence audit; no protocol-registered systematic review was performed. Clinical studies were heterogeneous and largely non-randomized; therefore, we did not attempt a pooled efficacy estimate.

**Results:**

Active and stable PD remain clinically useful categories, although their operational definitions vary across studies. Experimental PD models identify fibroblast-to-myofibroblast transformation as a pharmacologically accessible process, with PDE5 inhibitors and SERMs showing timing-dependent antifibrotic effects. Retrospective PDE5 inhibitor studies and clinical audits of PDE5 inhibitor plus tamoxifen therapy report mixed observations on progression and pain, but interpretation is constrained by non-randomized design, small or imbalanced controls, confounding, and heterogeneous endpoints. Placebo-controlled monotherapy evidence remains negative or neutral for tamoxifen, especially when disease duration is broad or not stratified by active inflammatory features. Guidelines and evidence reviews support individualized conservative care without endorsing PDE5 inhibitor/SERM therapy as standard practice.

**Conclusion:**

Active/progressive PD is a defensible setting for early antifibrotic trials. PDE5 inhibitor/SERM therapy is among the best-developed mechanistic candidates, but remains investigational. Clinical adoption requires prospective, randomized, stage-defined studies with standardized curvature assessment, plaque imaging, erectile-function measurement, pain assessment, co-intervention documentation, adverse-event capture, and PD-specific patient-reported outcomes.

## Introduction

Peyronie’s disease (PD) is often recognized through its anatomical consequences: penile curvature, shortening, indentation, hourglass deformity, erectile dysfunction, pain, difficulty with penetration, and sexual distress. The clinical problem usually begins earlier, during a period of changing deformity, pain, uncertainty, and anxiety, when plaque architecture may still be evolving.

The distinction between active and stable PD is useful in practice, but difficult to standardize. Contemporary intervention studies and reviews use inconsistent definitions of acute, active, chronic, and stable disease. Criteria vary by symptom duration, deformity stability, pain, or mixed definitions; one-third of primary intervention studies using phase labels did not define acute or chronic criteria.^1^ Recent International Consultation on Sexual Medicine (ICSM) recommendations and phase-definition reviews emphasize progressive deformity as a central feature of active disease and caution against pain alone as a phase marker, because pain can improve spontaneously or persist for mechanical reasons.^1^^,^^2^

This uncertainty has practical consequences for early pharmacological intervention. A regimen intended to slow active fibrosis cannot be evaluated fairly in a mixed population of early progressive disease, stable plaques, calcified plaques, and pain-only presentations. In the absence of a validated molecular staging system, trials need explicit clinical eligibility criteria and transparent endpoint selection.

Several recent Sexual Medicine Reviews publications have addressed phase definitions and clinical management recommendations in PD.^1^^,^^2^ Here, we focus on a narrower problem: whether active/progressive PD can serve as a clinically identifiable early antifibrotic trial window, using PDE5 inhibitor/selective estrogen receptor modulator (SERM) therapy as the leading candidate pharmacological model. We integrate phase-definition literature, antifibrotic primary-cell and animal pharmacology, early clinical observations, guideline context, outcome methodology, mechanical co-interventions, and omics evidence. The research question was: can active/progressive PD be defined well enough, clinically and biologically, to justify prospective testing of early antifibrotic pharmacotherapy, and what outcomes and design safeguards are required before PDE5 inhibitor/SERM therapy could move beyond an investigational model?

## Materials and methods

We used a narrative translational design. The working question was whether active or progressive PD provides a defensible setting for early antifibrotic pharmacological trials, and which clinical, mechanistic, outcome, and trial-design constraints should govern such studies.

We identified sources iteratively through targeted searches and citation chasing, with full-text checking of key sources when available. Searches and source updates were completed in May 2026. The main source types were PubMed/MEDLINE-indexed clinical, preclinical, guideline, and review articles; Cochrane and guideline evidence summaries; publisher full texts available through institutional or library access; and NCBI GEO or related public-data records for transcriptomic studies. Search concepts combined PD, penile induration, tunica albuginea, active phase, acute phase, stable phase, chronic phase, fibrosis, myofibroblast, TGF-beta, PDE5 inhibitor, tadalafil, sildenafil, vardenafil, tamoxifen, SERM, penile traction, vacuum device, patient-reported outcome, Peyronie’s Disease Questionnaire, transcriptomics, single-cell, GEO, and Dupuytren’s disease.

We prioritized full-text sources directly relevant to active/stable PD definitions; clinical studies of daily tadalafil, tadalafil-based combinations, and PDE5 inhibitor/SERM therapy; randomized placebo-controlled tamoxifen evidence; current guideline or consensus documents; non-surgical evidence reviews; primary-cell antifibrotic studies; drug-repurposing screens; patient-reported outcome measure validation or reliability studies; penile traction literature; and transcriptomic or single-cell studies in PD or related localized fibrosis. Surgical-only stable-phase studies were used only when needed for disease-stage context. Non-PD fibrosis literature was limited to biologically relevant cross-fibrosis comparators, particularly Dupuytren’s disease.

The review was narrative rather than systematic or formally scoping: no protocol was registered, no PRISMA flow diagram was constructed, and no formal risk-of-bias tool was applied. Because the clinical literature is heterogeneous, frequently retrospective, and often not stage-defined, we did not calculate pooled estimates or infer causality from observational data. We classified evidence by evidentiary role: clinical observations relevant to early pharmacological intervention (Table 1), mechanistic support for druggability of myofibroblast transformation (Table 2), and claim calibration for reviewers (Table 3). No original patient-level data, device-derived measurements, imaging assessments, laboratory measurements, or statistical software analyses were generated; institutional review board approval and informed consent were therefore not applicable.

**Table 1:** Clinical evidence relevant to early pharmacological intervention in active Peyronie’s disease.

**Study**	**Design and population**	**Intervention**	**Comparator**	**Key signal**	**Main limitation**	**Interpretation**
Spirito *et al.**^3^*	*Retrospective comparative study; acute PD with erectile dysfunction; n = 191*	*Tadalafil 5 mg daily; n = 108*	*Observation among patients who refused tadalafil; n = 83*	*Lower curvature progression at 12 weeks; stronger association when disease duration was 9 months or less; 5-item International Index of Erectile Function (IIEF-5), Peyronie’s Disease Questionnaire (PDQ)-Overall, and PDQ-Penile Pain improved*	*Non-randomized allocation; baseline imbalances; ED population; completer analysis*	*Supports interest in early PDE5 inhibition, not causal proof*
Durukan *et al.**^4^*	*Retrospective cohort; active PD; n = 133*	*Daily PDE5 inhibitor; n = 101; mostly sildenafil 25 mg, tadalafil 5 mg in 2 patients*	*No treatment; n = 32*	*No significant adjusted effect on curvature progression; shorter pain duration*	*Retrospective; non-randomized; natural-erection self-photography; possible ED and adherence limitations*	*Counter-signal for curvature modification; supports pain-focused uncertainty*
Dell’Atti and Slyusar*^5^*	*Retrospective cohort; active PD with erectile dysfunction; n = 161*	*Tadalafil 5 mg daily plus acetylsalicylic acid; n = 68*	*Tadalafil 5 mg daily; n = 93*	*Lower curvature and pain at 6 months in the combination group*	*Non-randomized; no placebo or untreated control; aspirin use largely reflected cardiovascular indications; all patients received tadalafil*	*Additional observation for tadalafil-based combination care, confounded by aspirin indication*
Shah *et al.**^6^*	*Clinical audit; acute PD without ED; n = 133 intervention and n = 26 comparison*	*PDE5 inhibitor plus tamoxifen for 3 months*	*No treatment or vitamin E at another hospital*	*Curvature improved in 42.9% vs 15.4%; curvature worsened in 9.0% vs 30.8%; pain decreased more in the treated cohort*	*Not randomized or blinded; small comparison group; different hospital settings; ED and traction/VED users excluded; outcome assessment not centrally standardized*	*Strongest current clinical observation for PDE5i/SERM therapy; efficacy remains unproven*
Ralph *et al.**^7^*	*Preliminary uncontrolled tamoxifen study; n = 36*	*Tamoxifen 20 mg twice daily for 3 months*	*None*	*Greatest response reported in disease less than 4 months and in patients with inflammatory infiltrate; chronic presentations did not show a comparable signal*	*Uncontrolled, preliminary, old study; not definitive efficacy evidence*	*Supports timing-dependent inflammatory-window hypothesis*
Cellek *et al.**^8^*	*Commentary/first clinical signal after abstract presentation*	*PDE5 inhibitor plus tamoxifen concept*	*Early non-randomized experience*	*Preliminary rationale supporting combined clinical testing*	*Opinion-level evidence, now superseded for clinical details by Shah et al.* *^6^*	*Useful translational context, not efficacy evidence*
Geelhoed *et al.**^9^*	*Retrospective off-label series; acute PD; n = 48*	*Tamoxifen plus tadalafil/vardenafil with concurrent vacuum erection device*	*None*	*Curvature, pain, intercourse, and erection improvements reported; adverse reactions in 39.6%*	*No untreated control; VED confounds attribution; self-reported outcomes; no validated PROMs; retrospective*	*Feasibility observation; cannot isolate drug effect*
Teloken *et al.**^10^*	*Randomized placebo-controlled trial; n = 25; non-calcified plaques; disease duration 4-72 months, mean about 20 months*	*Tamoxifen 20 mg twice daily for 3 months*	*Placebo*	*No significant advantage for pain, subjective deformity, plaque area, or curvature angle*	*Small, old, short trial; broad disease duration extending into a chronic/stable range; not modern active-phase stratified*	*Essential counterweight: tamoxifen monotherapy is not established therapy*

Abbreviations: PD, Peyronie’s disease; SERM, selective estrogen receptor modulator; VED, vacuum erection device.

**Table 2:** Mechanistic evidence supporting druggability of myofibroblast transformation.

**Study**	**Model**	**Intervention or drug class**	**Main finding**	**Translational interpretation**	**Limitation**
Ilg *et al.*^11^	*Human PD fibroblast models and rat PD model*	*PDE5 inhibitors and SERMs; vardenafil plus tamoxifen*	*Combination inhibited myofibroblast transformation beyond arithmetic additivity in vitro; combination also improved selected rat-model fibrosis readouts more than either drug alone*	*Core preclinical rationale for simultaneous PDE5i/SERM testing in active disease*	*Preclinical; not clinical efficacy*
Ilg *et al.*^12^	*Primary human tunica albuginea fibroblasts stimulated with transforming growth factor beta 1 (TGF-beta1)*	*Vardenafil and tamoxifen*	*Prevented but did not reliably reverse established myofibroblast traits*	*Supports an early-window model and argues against late plaque reversal*	*In vitro timing cannot be mapped directly to symptom duration*
Ilg *et al.*^13^	*Primary human PD fibroblasts stimulated with TGF-beta1; temporal fibrosis-panel expression assay*	*Gene-expression sampling at 0, 24, 36, 48, and 72 hours*	*Most gene-expression changes occurred within 24 hours and persisted; mitogen-activated protein kinase (MAPK)-Hedgehog pathway changes were observed at 36 hours*	*Supports possible molecular timing boundary and point-of-no-return biology*	*Limited to 760-gene fibrosis panel; in vitro timing cannot define clinical duration cut-offs*
Ilg *et al.*^14^	*Phenotypic screen of 1,953 US Food and Drug Administration (FDA)-approved drugs in primary human PD fibroblasts*	*FDA-approved drug library*	*Identified 26 candidate hits using alpha-SMA and viability readouts*	*Drug repurposing is feasible in PD-relevant primary-cell assays*	*Primary screen used one concentration and one patient-derived line; requires validation and clinical translation*
Harding *et al.*^15^	*Primary human tunica albuginea fibroblasts*	*PDE1/PDE4 inhibitors; lower-potency PDE7/PDE8 signals*	*Prevented myofibroblast transformation, apparently through cAMP-related mechanisms*	*Extends druggable PDE biology beyond PDE5*	*Preclinical only*
Ilg *et al.*^16^	*Primary human PD fibroblasts*	*Statins, PDE5 inhibitors, SERMs, combinations*	*Statins inhibited transformation; simvastatin synergized with vardenafil but not tamoxifen*	*Combination biology is selective; not all antifibrotic combinations synergize*	*In vitro; mechanism and clinical safety unknown*

Abbreviations: ESSM, European Society for Sexual Medicine; PD, Peyronie’s disease; SERM, selective estrogen receptor modulator.

**Table 3:** Claim calibration for reviewers.

**Statement**	**Current support**	**Evidence base**	**Conservative interpretation**
*Active/progressive PD is biologically dynamic*	*Supported conceptually*	*Phase-definition literature, guidelines, mechanistic and omics studies* ^1^ ^,^ ^2^^,^^11–13^^,^^17–21^	*Reasonable framework for trial design, not a validated biomarker stage*
*Fibroblast-to-myofibroblast transformation is druggable in PD models*	*Supported preclinically*	*Primary-cell, animal, temporal-expression, and drug-screening models* ^11-16^	*Strong biological rationale, not proof of patient benefit*
*PDE5i/SERM therapy has early clinical observations in active PD*	*Preliminary and unproven*	*Early clinical observations* ^6^ ^,^ ^8^^,^^9^	*Prospective-testing rationale; investigational and off-label*
*Daily PDE5 inhibitor therapy may reduce progression in selected active PD patients*	*Mixed and non-definitive*	*Retrospective PDE5 inhibitor studies* ^3-5^	*Possible benefit, especially for pain or ED-related outcomes, but confounded*
*Tamoxifen monotherapy is established treatment*	*Not supported*	*Preliminary timing signal and placebo-controlled counterweight* ^7^ ^,^ ^10^	*Should not be claimed*
*Current guidelines endorse PDE5i/SERM as standard care*	*Not supported*	*Recommendations and evidence reviews* ^2^ ^,^ ^22–24^	*Current guidelines support individualized conservative care, not this combination as standard*
*Traction can be part of conservative multimodal management*	*Plausible but low-certainty and device-specific*	*Guidelines, ESSM position statements, RestoreX data* ^2^ ^,^ ^25^^,^^26^	*Must be separated from systemic drug efficacy in trials*
*Public omics can prove treatment efficacy*	*Not supported*	*PD and Dupuytren public-data studies* ^13^ ^,^ ^18–21^	*Exploratory only*

Abbreviations: ESSM, European Society for Sexual Medicine; PD, Peyronie’s disease; SERM, selective estrogen receptor modulator.

## Results

### Active PD as an unstable but useful clinical construct

Stage-based language is embedded in PD care. Active disease generally implies progressive or evolving deformity, often with pain; stable disease generally implies no meaningful deformity change over a defined period and is the usual context for corrective procedures. Phase definitions in the literature remain inconsistent.^1^

**Figure 1:**
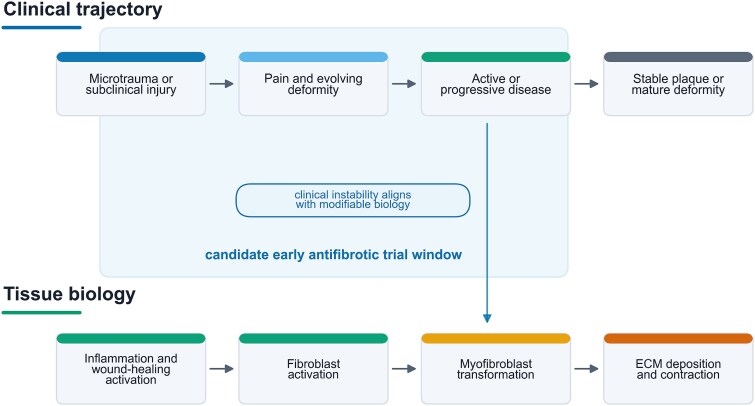
Active Peyronie’s disease as inflammatory-fibrotic remodeling. Conceptual figure showing a clinical trajectory from microtrauma or subclinical injury through pain and evolving deformity, active or progressive disease, and stable plaque or mature deformity; linked tissue biology from inflammation and wound-healing activation through fibroblast activation, myofibroblast transformation, extracellular matrix deposition, and contraction; and the proposed early antifibrotic trial window.

In future antifibrotic trials, active PD is best treated as an eligibility construct. A defensible definition would report time from first symptom and first deformity, documented or clearly reported curvature progression, pain status and trajectory, plaque palpation and ultrasound phenotype, calcification status, erectile function, prior therapy, and evidence for stability or instability over a prespecified interval. Such reporting would allow investigators to test which clinical features predict response. Figure 1 summarizes the proposed clinical-biological trajectory and early antifibrotic trial window.

### Fibroblast-to-myofibroblast transformation as a therapeutic target

PD pathobiology is commonly linked to repetitive microtrauma or subclinical injury followed by abnormal wound healing. Transforming growth factor beta signaling, extracellular matrix deposition, oxidative stress, inflammation, collagen remodeling, and fibroblast-to-myofibroblast transformation recur across experimental and review literature.^2^^,^^11^^,^^12^^,^^17^ These pathways overlap with Wnt/beta-catenin, platelet-derived growth factor, NF-kB, JAK–STAT, MAPK, RhoA/ROCK, MMP/TIMP imbalance, apoptosis/autophagy, oxidative stress, and immune-cell signaling.^17^

Myofibroblasts are central to this model because they combine synthetic and contractile functions. They produce extracellular matrix, contribute to tissue contraction, and can convert a dynamic reparative response into persistent plaque architecture. Peyronie’s disease progression therefore reflects a tissue-state change, with collagen accumulation as one component rather than the whole process.

**Figure 2:**
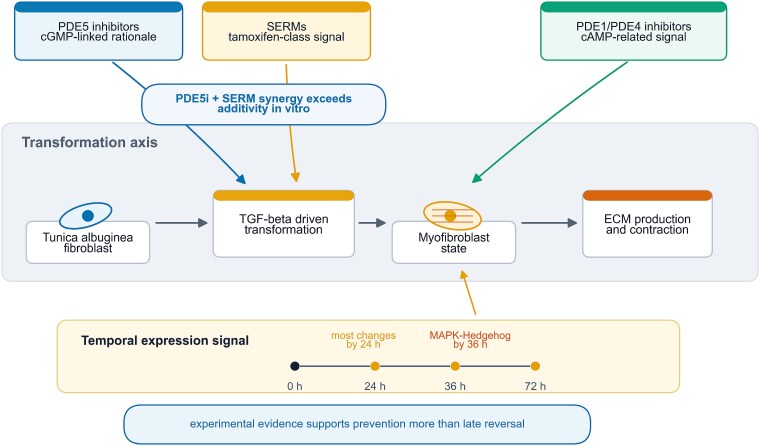
Drugging myofibroblast transformation in Peyronie’s disease. Mechanistic figure showing fibroblast-to-myofibroblast transformation, TGF-beta-driven induction, the myofibroblast state, extracellular matrix production, and contraction, with candidate drug classes including PDE5 inhibitors, selective estrogen receptor modulators (SERMs), and PDE1/PDE4 inhibitors. It distinguishes prevention of myofibroblast transformation in experimental systems from the lower apparent reversibility of established transformation, shows that PDE5 inhibitor/SERM synergy exceeded arithmetic additivity in vitro, and notes temporal-expression evidence relevant to possible irreversibility.

The available models make timing central. Primary human tunica albuginea fibroblast models show that PDE5 inhibition and SERM treatment can prevent transforming growth factor beta-induced ASMA/ACTA2-positive myofibroblast transformation, but do not reverse established ASMA expression or extracellular matrix production after prolonged transforming growth factor beta exposure; tamoxifen affected collagen contraction only.^12^ A subsequent temporal gene-expression study in TGF-beta1-stimulated primary PD fibroblasts found that most expression changes occurred within the first 24 hours and persisted through 72 hours, with MAPK-Hedgehog pathway changes at 36 hours, supporting a possible point-of-no-return model while remaining limited to a 760-gene fibrosis panel.^13^ Together, these data make early intervention the testable hypothesis, although they do not define a clinical cut-off such as 3, 6, 9, or 12 months. A realistic trial endpoint would be reduction of progression, pain, and functional loss; mature plaque dissolution is unlikely to be the relevant target. The candidate timing model for drugging myofibroblast transformation is shown in Figure 2.

### Antifibrotic pharmacology and drug repurposing

The PDE5 inhibitor/SERM concept emerged from disease-relevant phenotypic screening and antifibrotic assays. In experimental PD systems, PDE5 inhibitors and SERMs inhibited myofibroblast transformation. The combination signal goes beyond additivity in vitro: when vardenafil and tamoxifen were tested together, inhibition of TGF-beta1-induced myofibroblast transformation exceeded the arithmetic sum of either compound alone. Vardenafil plus tamoxifen also inhibited collagen gel contraction and extracellular matrix production, and showed greater activity on selected molecular and histologic fibrosis readouts than either class alone in a transforming growth factor beta rat model that does not reproduce penile curvature.^11^ This synergy gives a biological rationale for simultaneous combined testing, while falling short of clinical efficacy proof.

Subsequent work sharpened the timing argument by showing prevention more convincingly than reversal of established myofibroblast transformation.^12^ Other drug-repurposing studies have expanded the candidate list: a phenotypic screen of 1953 FDA-approved drugs identified candidate hits in primary human PD fibroblasts, although the primary screen used one drug concentration and one patient-derived line; PDE1/PDE4 inhibition can prevent myofibroblast transformation, with weaker effects from PDE7/PDE8 inhibition; and statins appear antifibrotic in vitro, with simvastatin synergizing with vardenafil but not tamoxifen.^14–16^ PDE biology extends beyond canonical PDE5-cGMP signaling: PDE1/PDE4 inhibition increased cAMP in tunica albuginea-derived fibroblasts, and transforming growth factor beta exposure downregulated PDE5A, supporting a time-dependent shift in drug target availability.^12^^,^^15^ Together, these studies argue that PD fibroblast biology is pharmacologically tractable and that combination effects are selective; statin/PDE5 inhibitor combinations also require clinical safety caution.

### Clinical observations for early pharmacological intervention

The principal clinical studies informing early pharmacological intervention are summarized in Table 1.

Daily tadalafil has been evaluated in a retrospective comparative study of men with acute-phase PD and erectile dysfunction. Men who accepted tadalafil 5 mg daily had lower curvature progression at 12 weeks than men managed by observation, with a stronger association when disease duration was 9 months or less; IIEF-5, PDQ-Overall, and PDQ-Penile Pain also improved.^3^ A separate retrospective cohort found no significant adjusted effect of daily PDE5 inhibitors, mostly sildenafil 25 mg, on curvature progression, but reported shorter pain duration.^4^ Another retrospective cohort reported lower curvature and pain at 6 months with tadalafil plus acetylsalicylic acid compared with tadalafil alone, although the acetylsalicylic acid group largely reflected pre-existing low-dose aspirin use for cardiovascular indications.^5^ These studies keep clinical interest alive, although treatment allocation was non-randomized, baseline imbalances and nonblinding were common, prior or concurrent erectile dysfunction may affect patient-reported outcomes, and curvature assessment can vary by technique.

The most informative recent clinical dataset for PDE5 inhibitor/SERM therapy is the full Shah et al. clinical audit of men with acute PD treated for 3 months with off-label PDE5 inhibitor plus tamoxifen, compared with no treatment or vitamin E at another hospital. Curvature improved in 42.9% of the combination group and 15.4% of the comparison group; worsening occurred in 9.0% and 30.8%, respectively. Pain decreased in both cohorts, but the decrease was larger with PDE5 inhibitor plus tamoxifen.^6^ The findings fit the preclinical timing model, but the design leaves important uncertainty: treatment was not randomized or blinded, cohorts came from different hospital settings, the comparison group was small, men with erectile dysfunction and traction or vacuum-device use were excluded, adverse-event inference was limited, and curvature and plaque assessment were not centrally standardized. The earlier commentary that followed the first clinical signal is best treated as translational context now that the full audit has been published.^8^ A separate retrospective off-label series supports feasibility of tamoxifen plus tadalafil or vardenafil in acute-phase care, although pharmacological effects cannot be isolated because vacuum erection device use was included.^9^

Tamoxifen monotherapy provides a timing signal and a cautionary clinical counterweight. In an earlier preliminary study, tamoxifen 20 mg twice daily for 3 months was associated with improvement in subsets of patients, with the greatest response reported in disease of less than 4 months’ duration and in patients with inflammatory infiltrate; chronic presentations did not show a comparable signal. This supports an acute inflammatory-window hypothesis, but the study was uncontrolled and cannot establish efficacy.^7^ By contrast, a later randomized placebo-controlled trial enrolled men with non-calcified plaques but broad disease duration extending into a chronic/stable range, from 4 to 72 months with a mean of approximately 20 months, and did not significantly outperform placebo for pain, subjective deformity, subjective plaque-size change, objective plaque area, or curvature angle.^10^ These studies argue against tamoxifen as established monotherapy while reinforcing the importance of symptom duration, inflammatory activity, and stage definition in future combination trials.

### Guideline context, traction, and patient-reported outcomes

Current recommendations recognize active inflammatory disease and individualized management, while stopping short of establishing oral therapy as a proven method for meaningful deformity reduction. The 2024 International Consultation on Sexual Medicine recommendations allow daily PDE5 inhibitors in patients with concomitant erectile dysfunction or penetrative difficulty and recognize penile extender devices and vacuum devices as potential deformity-reducing strategies in selected contexts.^2^ The American Urological Association guideline remains an important cautionary source for oral therapies, and the European Association of Urology update provides current European terminology and management context.^22^^,^^23^ The Cochrane review of non-surgical therapies emphasizes limited certainty for many conservative treatments and the need for better trials; it is an evidence-quality anchor for nonsurgical PD therapy generally, not direct randomized evidence for PDE5 inhibitor/SERM therapy.^24^

Mechanical remodeling needs to be separated from systemic drug efficacy. European Society for Sexual Medicine position statements consider penile traction therapy promising but limited by heterogeneous low-certainty evidence. RestoreX provides device-specific evidence for objective length and curvature improvement through a 6-month open-label phase with short daily use, plus 9-month self-reported continued use and benefit; these data should not be generalized to all traction devices or to vacuum devices.^25^^,^^26^ Recent multimodal proposals illustrate the field’s movement toward integrated pharmacologic, biologic, and mechanical strategies, and also show why component effects need to be separated. The 3Ts article is best read as a structured protocol proposal; it does not show that tadalafil, biologics, or traction independently modify PD fibrosis.^27^ If traction or vacuum therapy is included in future pharmacological studies, device type, prescribed duration, adherence, adverse effects, and co-intervention rules need to be standardized and reported.

Outcome selection will determine whether a trial can be interpreted. The Peyronie’s Disease Questionnaire has construct-validity and test–retest evidence for psychological and physical symptoms, penile pain, and symptom bother, but its validation base is largely sexually active chronic or stable PD.^28^^,^^29^ The PEYROnie’s disease Questionnaire (PEYRO-Q) is a preliminarily validated multidimensional PD-specific patient-reported outcome measure that may broaden assessment, although responsiveness, minimal clinically important difference, cross-cultural performance, and direct comparison with PDQ remain unresolved.^30^ Active-phase trials should pair objective deformity and plaque imaging with pain, erectile function, penetrative ability, treatment adherence, adverse events, and PD-specific patient-reported outcomes.

### Omics and cross-fibrosis evidence

Temporal omics evidence now links the prevention-not-reversal pharmacology to a possible molecular timing boundary. In primary human PD fibroblasts stimulated with TGF-beta1, most gene-expression changes in a fibrosis-panel assay occurred within the first 24 hours and persisted through 72 hours; by 36 hours, MAPK-Hedgehog pathway changes were detected.^13^ The assay cannot set a clinical time cut-off, but it supports the idea that early active disease may be biologically different from established plaque biology.

Single-cell and histologic work in stable chronic surgical PD plaques supports a more complex interpretation than passive scar tissue. Peyronie’s disease tissue showed fibroblast, myofibroblast, smooth muscle, endothelial, myeloid, and T-cell populations, with monocyte-derived myeloid cells and cytotoxic T-cell or mucosal-associated invariant T-cell states consistent with persistent immune activation. Neutrophils were not a PD-enriched population.^18^ These data are limited by very small single-cell sample size and chronic surgical context and cannot directly define active-phase drug-response biology.

Fibroblast RNA-seq and integrated public-data analyses provide complementary exploratory evidence. GSE146500 is a small cultured-fibroblast perturbation dataset comparing normal fibroblasts, PD fibroblasts, and PD fibroblasts treated with pericyte-derived extracellular vesicle-mimetic nanovesicles; it is not PDE5 inhibitor/SERM evidence.^19^ GSE126005 and GSE146500 have already been integrated in a hub-gene analysis identifying cytokine and Janus kinase–signal transducer and activator of transcription (JAK–STAT) pathway activity.^20^ Future work could go beyond simple differential expression by integrating PD disease signatures, related fibrosis cell states, and drug-perturbation resources. Dupuytren’s disease is a relevant comparator model because a single-cell atlas has identified inflammatory fibroblast and myofibroblast states in localized human fibrosis.^21^

## Discussion

### What the current evidence supports

The current literature supports a mechanistically grounded model that still needs clinical testing. Active/progressive PD is clinically meaningful, biologically dynamic, and consistent with inflammatory-fibrotic remodeling. Peyronie’s disease fibroblast models show that myofibroblast transformation can be pharmacologically inhibited, and PDE5 inhibitor/SERM combinations have a disease-relevant preclinical rationale. Early clinical studies report mixed observations on progression or pain, and remain retrospective, observational, or audit-based.

**Figure 3:**
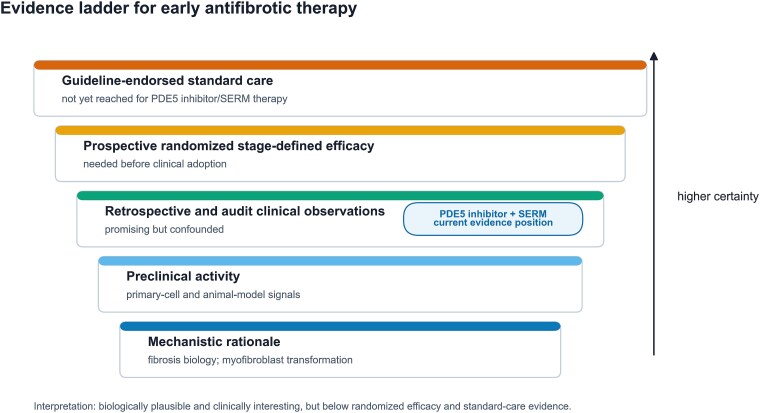
Evidence ladder for early antifibrotic therapy. Evidence-ladder figure distinguishing mechanistic rationale, preclinical activity, retrospective clinical observations, prospective observational evidence, randomized efficacy evidence, and guideline-endorsed standard care. PDE5 inhibitor/selective estrogen receptor modulator therapy and PDE5 inhibitor-based strategies are placed as mechanistically grounded with early clinical observations, while clearly below randomized efficacy evidence or guideline-endorsed standard care.

Current evidence justifies prospective testing of PDE5 inhibitor/SERM therapy; routine adoption would be premature. The same applies to tadalafil-based therapy: current data are most suggestive in men with erectile dysfunction and early disease, but they do not prove disease modification. Figure 3 places these observations on a translational evidence ladder.

Differences among the available clinical observations are expected because the studies do not estimate the same treatment effect. Spirito et al. evaluated tadalafil 5 mg daily in acute PD with erectile dysfunction and short-term follow-up; Durukan et al. evaluated mixed daily PDE5 inhibitor use, mostly sildenafil 25 mg, in a broader active-phase cohort and found no significant adjusted curvature effect but shorter pain duration; Dell’Atti and Slyusar compared tadalafil plus acetylsalicylic acid with tadalafil alone, making aspirin indication and vascular comorbidity important confounders; and Shah et al. evaluated PDE5 inhibitor/SERM therapy in men without erectile dysfunction or traction/vacuum-device use, with a small comparison cohort from another hospital.^3–6^ These differences in population, drug class and dose, erectile-function status, co-interventions, comparator structure, follow-up, and curvature measurement explain why the literature should not be read as a simple positive-vs-negative vote.

### Boundaries of interpretation

At present, PDE5 inhibitor/SERM combination therapy should remain investigational rather than standard care. Tamoxifen monotherapy also lacks established efficacy. Evidence for reversal of mature, stable, or calcified plaques is absent, and public omics or in vitro drug response remain hypothesis-generating rather than efficacy evidence.

The distinction matters clinically. Active-phase pain may improve naturally, subjective and objective deformity measures can correlate poorly, erectile-function treatment can change sexual function and measurement conditions, and curvature assessment can vary with erection quality and technique.^2^^,^^22^ Erectile dysfunction status is both a treatment indication and a potential outcome confounder in tadalafil studies, whereas the recent PDE5 inhibitor/SERM audit excluded men with erectile dysfunction.^3^^,^^6^ This difference limits direct generalization across study populations.

### Implications for future trials

A logical next step is a prospective, randomized, stage-defined trial. Inclusion criteria would require active or unstable disease, preferably documented by progressive deformity and not pain alone. Disease duration, deformity trajectory, plaque calcification, erectile function, prior therapy, and co-interventions need to be recorded and stratified.

Such a trial is difficult for reasons that are specific to acute PD. Recruitment must occur before stabilization, yet many patients present after symptoms have evolved for months; pain can improve naturally, making pain-only endpoints vulnerable to regression toward the mean; curvature measurement varies with erection induction, rigidity, photography, and assessor technique; and co-interventions such as traction, vacuum therapy, analgesics, erectile-function treatment, and patient preference for active treatment can compromise randomization and attribution.^6^ Randomized testing remains feasible if trials use explicit phase criteria, blinded standardized curvature assessment where feasible, prespecified co-intervention rules, and objective plaque imaging.

Comparator choice follows the estimand. If the objective is to isolate the pharmacological effect of PDE5 inhibitor/SERM therapy, traction and vacuum devices need to be excluded, balanced, or protocolized across groups. If the objective is pragmatic multimodal care, mechanical therapies need to be treated as active co-interventions with adherence measurement. Primary endpoints should prioritize objective curvature progression or change under standardized, pharmacologically induced erection conditions, with blinded assessment where feasible. Key secondary endpoints should include penile length, deformity descriptors, plaque ultrasound and calcification, pain during erection, erectile function, penetrative capacity, PD-specific patient-reported outcomes, adverse events, discontinuation, drug and device adherence, and rescue therapy or surgery.

### Limitations of this review

Because this is a narrative translational synthesis, it prioritizes the biological and clinical logic of early antifibrotic intervention over exhaustive treatment comparison. The source-selection strategy was targeted rather than systematic, so relevant studies may have been missed. The clinical evidence base is mostly retrospective, non-randomized, short-term, or observational. Control groups are often small, absent, or defined by treatment refusal. Co-interventions such as traction and vacuum therapy can confound interpretation. Preclinical findings are disease-relevant, but they often use surgical or non-plaque tunica albuginea cells and transforming growth factor beta stimulation, and cannot reproduce the mechanical, vascular, neural, sexual, and inflammatory complexity of living PD. Omics evidence remains exploratory and is mostly derived from chronic surgical samples or cell-culture models.

## Conclusion

Active/progressive PD remains a defensible target for early antifibrotic trials. We frame that target as a clinically definable fibrotic window that is ready for prospective testing, while keeping PDE5 inhibitor/SERM therapy in its proper investigational position. Practice should not change without prospective validation. The field now needs prospective, blinded, stage-defined studies that combine objective deformity assessment, imaging, erectile-function measurement, pain assessment, adverse-event capture, co-intervention documentation, and PD-specific patient-reported outcomes.

## Data Availability

No original datasets were generated for this review. Public datasets and resources discussed in the manuscript are cited in the text where relevant. No new data were generated or analyzed in support of this review.
